# Supplementary material for: Charge Carrier Relaxation in Colloidal FAPbI3 Nanostructures Using Global Analysis
Source: Nanomaterials (Basel). 2020 Sep 23;10(10):1897. doi: 10.3390/nano10101897 (PMC7598295; doi:10.3390/nano10101897)
Supplement: Supplementary file 1 [file nanomaterials-10-01897-s001.pdf]

Supplementary Materials

Charge Carrier Relaxation in Colloidal $FAPbI_3$ Nanostructures Using Global Analysis

Carolina Villamil Franco [1], Benoît Mahler [2], Christian Cornaggia [1], Thomas Gustavsson [1] and Elsa Cassette [1,*]

[1] Université Paris-Saclay, CEA, CNRS, Laboratoire Interactions, Dynamiques et Lasers (LIDYL), F-91191 Gif-sur-Yvette, France; carolina.villamil-franco@cea.fr (C.V.F); christian.cornaggia@cea.fr (C.C); thomas.gustavsson@cea.fr (T.G)

[2] Université de Lyon, Université Claude Bernard Lyon 1, CNRS, Institut Lumière Matière (ILM), F-69622 Villeurbanne, France; benoit.mahler@univ-lyon1.fr (B.M)

* Correspondence: elsa.cassette@cea.fr (E.C)

1. Material Synthesis and Characterizations

1.1. Chemical List.

Lead iodide (PbI_2 99.999%), formamidinium iodide (FA-I, anhydrous, > 99%), formamidinium acetate (FA-acetate, 99%), N,N-dimethylformamide (DMF, anhydrous, 99.8%), chloroform (anhydrous, 99%), toluene (anhydrous, 99.8%) and acetonitrile (anhydrous, 99.8%) were purchased from Sigma-Aldrich. Oleic acid (OA, 99%) and octadecene (ODE, 90%) were purchased from Alfa Aesar. Oleylamine (OLAm, 90%) was purchased from Acros Organics.

1.2. Synthesis of $FAPbI_3$ NCs via the "Hot Injection" Method.

A solution of PbI_2 was prepared by dissolving under argon at 80°C 0.187 mmol of PbI_2 in a mixture of vacuum-dried ODE (5 mL), OA (1 mL) and OLAm (0.5 mL). FA-oleate (~0.25 M) was prepared by dissolving FA-acetate (5 mmol) in a mixture of ODE/OA (16/4 mL, vacuum-dried) at 130°C under argon. The mixture was then dried under vacuum for 30 min at 50°C. The solution was preheated at 100°C under argon before use to avoid precipitation. 2 mL of the FA-oleate solution were introduced in a 3-neck flask at 100°C under argon and 6.5 mL of the PbI_2 solution was injected. After 1 min, the reaction is quenched with an ice bath. The resultant solution was centrifuged for 10 min at 13000 rpm. The supernatant was discarded and the precipitate re-dispersed in toluene. The NCs solution was then centrifuged for 5 min at 3000 rpm and just the supernatant was kept to eliminate large aggregates. No further washing step was employed.

1.3. Synthesis of Thick $FAPbI_3$ NPs via the LARP Method.

Solutions of PbI_2 and FA-I at 0.2 M in DMF were prepared by dissolving 0.2 mmol of each salt in 1 mL of DMF. At room temperature and ambient atmosphere, 125 μL of the PbI_2 solution at 0.2 M was mixed with 125 μL of the FA-I solution at 0.2 M. Then, the ligands OA (50 μL) and OLAm (40 μL) were added. This mixture (~340 μL) was injected into 15 mL of chloroform under strong stirring. The crude solution was separated in 1 mL aliquots and chloroform/acetonitrile mixture (100 μL, ratio 1:1) was added for precipitation. The solution was centrifuged at 5.0 rcf for 20 min. Then, the supernatant was discarded and the precipitate re-dispersed in chloroform to reach the adequate optical density for the experiments.

2. TA setup and data treatments.

2.1. Setup Description.

The pump beam was further modulated at 1.5 kHz by an optical chopper (Newport 3502). A broadband white light (WL) continuum probe beam was generated in a thin Sapphire plate using a fraction of the other part of the fundamental beam previously delayed with a motorized translation stage (60 cm, minimum step 200 nm). This motorized translation stage (M-IMS600CCHA, XPS controller, Newport) and mechanical shutters in the pump and probe arms were directly controlled with a home-made LabVIEW program. After WL generation, the fundamental was filtered and the WL power reduced to a few nJ. Before the sample, the pump power was controlled using a variable neutral density filters. The polarization of the pump and probe beam were set vertical. The probe and pump beams were focalized and overlapped at the center of the flow cell (quartz, 1 mm path length). The transmitted probe beam was recollimated and the successive probe spectra with pump ON and OFF were registered at 3 kHz by a commercial spectrograph (IsoPlane 160, Princeton Instruments) coupled with a CCD camera (ProEM-HS 1024B, Princeton Instruments). The CCD camera was indirectly controlled by LabVIEW via LightField software (Princeton Instruments). In order to measure a spectrum of 1024 pixels at 3 kHz with the CCD camera (3000 frames/s), we used a fast custom acquisition mode with a vertical binning of 64 pixels (13 μm each) over the region of interest (ROI) closest to the chip and an exposure time of 1 μs. A TTL signal at 3 kHz extracted from the laser electronics was used as the reference for the electronics of the TA apparatus, to synchronize the frequency of the optical chopper in the pump arm, to trig a low jitter digital delay generator (Stanford Research Systems DG645) and a gate integrator (Stanford Research Systems SR250). The delayed TTL signal was used to trig the CCD camera to measure WL spectra at 3 kHz (shot to shot). The timing was set in order that the WL pulse arrives on the CCD during the exposition time. The gate integrator was used to integrate the intensity of each pump pulse through a photodiode (PD, 200-1100 nm, Thorlabs DET10A/M), at 3 kHz, in order to 1) register the pump fluctuations (output voltage proportional to the PD intensity integrated over the gate) and 2) to identify ON and OFF pump pulses (pump at 1.5 kHz). Its output was sent to a DAQ card (National Instruments USB-6211, 250 kS/s). This DAQ card was trigged by a TTL signal of the CCD corresponding to the exposure time (3 kHz, rising edge).

2.2. TA Signal and IRF.

The TA signal was calculated as follow:

$$\Delta A(\lambda,t) = -log\left(\frac{1}{n}\sum_{k=0}^{m-1}\frac{I_{2k}^{ON}(\lambda,t) - I_{fluo}(\lambda)}{I_{2k+1}^{OFF}(\lambda,t) - I_{dark}(\lambda)}\right)$$

with $I(\lambda,t)$ the WL spectrum measured at a pump-probe time delay t with the pump ON for a pulse $2k$ (even) and the pump OFF for the following pulse $2k+1$ (odd), I_{fluo} the spectrum recorded with the probe beam blocked, I_{dark} the spectrum measured with both pump and probe blocked and m the number of ON/OFF kinetic cycle pairs to average at one position of the translation state (for each different t). Both I_{fluo} and I_{dark} were collected just before the scan collection, averaged over 3000 pulses. The sensitivity of the measured ΔA signal is about 2×10^{-4} (optical density) for $m = 250$, in the optimum wavelength range (480–740 nm).

The number of measured scans was typically varied from 1 to 5 for excitation fluency dependency measurements due to the finite stability of the recirculating sample. The temporal resolution is estimated to 130 fs from solvent response measurement (*i.e.* cross-phase modulation, Figure S1). At low excitation fluence, the TA signal is directly proportional to the pump power. In this linear regime, ΔA can be further corrected by the pump fluctuation (shot-to-shot or along the scan over T).

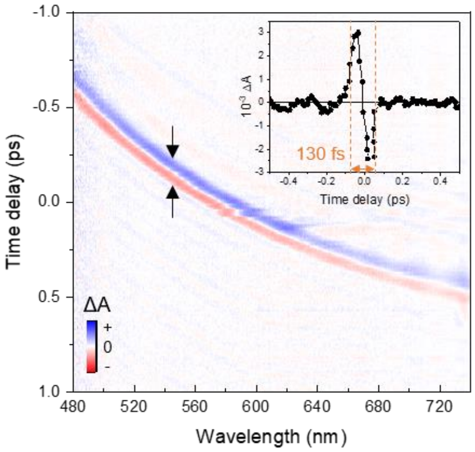

Figure S1. TA map of the solvent response (toluene). The chirp of the WL is clearly seen by the change of the position of $t = 0$ with the wavelength. At a given wavelength, the width of the non-resonant signal gives an estimation of the temporal resolution. An average over the wavelength (chirp corrected) gives the graph in inset, with a signal within 127 fs.

2.3. Chirp Correction of the TA Data.

The TA data consists in averaging the datasets, removing the static scatter (signal at $t < 200$ fs) and correct for the group velocity dispersion (chirp) of the white light to extract the accurate TA spectra for $t < 1$ ps (see Figure S2). Glotaran software [1] was used with a sequential model to fit the data with 2–3 exponential kinetic/spectral parameters, Gaussian IRF parameters and polynomial dispersion (2nd order). A coherent artifact might be added in the model to well reproduce the data at high excitation fluence.

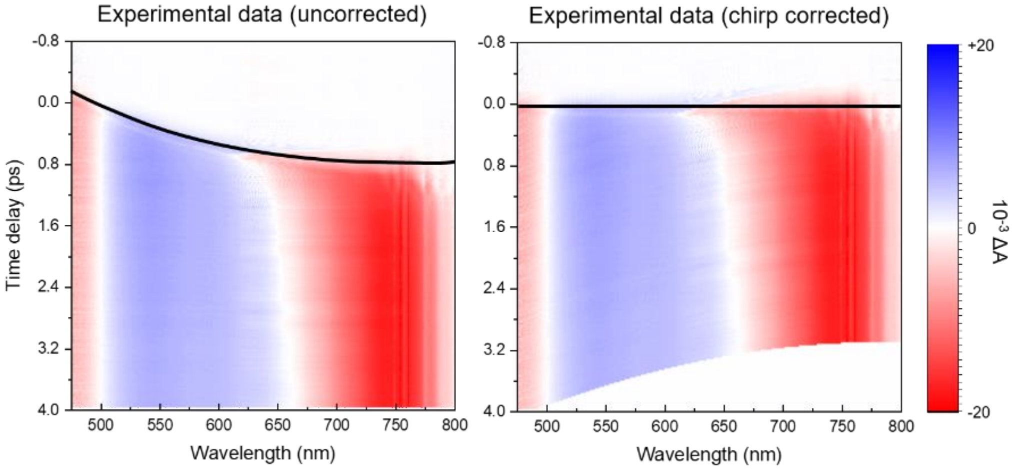

Figure S2. Plot of the TA maps of $FAPbI_3$ NPs excited at 630 nm, before (left) and after (right) chirp correction. The black lines is a visual aid corresponding to $t = 0$.

3. Calculation of the initial electron-hole pair density.

The average number of electron-hole pairs created per pump pulse per nanoparticle is:

$$\langle N \rangle = \frac{E_{pulse}\ \sigma_{abs}}{h\nu\ \Sigma_{laser}}$$

where E_{pulse} is the pump pulse energy, σ_{abs} is the absorption cross section of the material at the pump photon energy $h\nu$ and $\Sigma_{laser} = \pi(\omega_0)^2$ is the effective section of the pump laser beam with a beam waist ω_0 at the focus position. We have $E_{pulse} = P_0/RR$ with RR, the pump repetition rate (1.5 kHz) and P_0, the average power of the pump beam (from 5 μW to 1 mW). In order to account for the carrier-

carrier interactions, we need to consider the exciton density $\tilde{n} = N/V_{NP}$ in cm^{-3}, where V_{NP} is the nanoparticle volume.

Away from the confinement effects, the absorption cross section should scale linearly with the volume of the nanoparticle. This was confirmed for perovskite in cesium lead halide NCs at 3.6 eV (344 nm) [2]. Previously, Fang *et al.* have determined the absorption cross section of 13 nm cubic-shaped $FAPbI_3$ NCs to be 5.2×10^{-13} cm^2 at 400 nm [3]. We used this value to estimate the cross section of our $FAPbI_3$ samples at the different excitation energies (see below).

The volume estimated for the cubic NCs with $l = 12 \pm 2$ nm is $V_{NC} \approx 1970$ nm^3 and for the thick NPs (hexagonal shape) with lateral size $l = 70$ nm $\pm$ 20 nm and thickness $t = 11 \pm 3$ nm, the section is $S_{NP} \approx 3773$ nm^2 and the volume is $V_{NP} \approx 39925$ nm^3. Then, the deduced absorption cross section at 400 nm was $4.7 \mathrm{x} 10^{-13}$ cm^2 for the NCs and $9.4 \mathrm{x} 10^{-12}$ cm^2 for the thick NPs. From the absorption spectrum (of a diluted sample) which is proportional to the cross section, we get an absorption cross section of $4.7 \mathrm{x} 10^{-14}$ cm^2 for the cubic NCs at 650 nm and $1.2 \mathrm{x} 10^{-12}$ cm^2 ($3.6 \mathrm{x} 10^{-12}$) for the thick NPs at 630 nm (520 nm). The lowest number of electron-hole pairs created per pulse is $N_{NC} \approx 2.1$ (in term of density 5.5×10^{17} cm^{-3}) for an excitation of 13.4 μJ/cm^2 at 650 nm. For the thick NPs excited at 630 nm, $N_{NP} \approx 22$ (density 5.6×10^{17} cm^{-3}) and $N_{NP} \approx 224$ (density 5.6×10^{18} cm^{-3}) at 6 and 60 μJ/cm^2, respectively. We can note a larger value of N for the thick NPs compared to the cubic NCs because of the large difference of volume, while TA experiments were performed in the same range of ΔA (from few 10^{-3} to 10^{-1}). To study the carrier-carrier interactions, it is important to compare the exciton density $\tilde{n}$ rather than N.

REFERENCES

[1] Snellenburg, J.J.; Laptenok, S.P.; Seger, R.; Mullen, K.M.; Stokkun, I.H.M. Glotaran : A Java -Based Graphical User Interface for the R Package TIMP. *J. Stat. Softw.* **2012**, *49*, 1–22.
[2] Makarov, N.S.; Guo, S; Isaienko, O.; Liu, W.; Robel, I.; Klimov, V.I., Spectral and Dynamical Properties of Single Excitons, Biexcitons, and Trions in Cesium-Lead-Halide Perovskite Quantum Dots. *Nano Lett.* **2016**, *16*, 2349-2362.
[3] Fang, H.-H.; Protesescu, L.; Balazs, D.M.; Adjokatse, S.; Kovalenko, M.V.; Loi, M.A. Exciton Recombination in Formamidinium Lead Triiodide: Nanocrystals versus Thin Films. Small 2017, 13, 1700673.
